# Angiopoietin 2 as a Novel Potential Biomarker for Acute Aortic Dissection

**DOI:** 10.3389/fcvm.2021.743519

**Published:** 2021-12-23

**Authors:** Bi Huang, Li Tian, Zhaoran Chen, Liang Zhang, Wenjun Su, Tianyi Lu, Yanmin Yang, Rutai Hui, Xiaojian Wang, Xiaohan Fan

**Affiliations:** ^1^State Key Laboratory of Cardiovascular Disease, Fuwai Hospital, National Center for Cardiovascular Diseases, Chinese Academy of Medical Sciences and Peking Union Medical College, Beijing, China; ^2^Department of Cardiology, The First Affiliated Hospital of Chongqing Medical University, Chongqing, China; ^3^Department of Cardiology, the Second Affiliated Hospital of Harbin Medical University, Harbin, China; ^4^Department of Geriatrics and Gerontology, Beijing Friendship Hospital, Capital Medical University, Beijing, China

**Keywords:** transcriptome, angiopoietin 2, acute aortic dissection, biomarker, diagnosis

## Abstract

Biomarker-assisted diagnosis of acute aortic dissection (AAD) is important for initiation of treatment and improved survival. However, identification of biomarkers for AAD in blood is a challenging task. The present study aims to find the potential AAD biomarkers using a transcriptomic strategy. Arrays based genome-wide gene expression profiling were performed using ascending aortic tissues which were collected from AAD patients and healthy donors. The differentially expressed genes were validated using quantitative reverse transcriptase PCR (qRT-PCR) and western blot. The plasma levels of a potential biomarker, angiopoietin 2 (ANGPT2) were determined in case-control cohort (77 AAD patients and 82 healthy controls) by enzyme linked immunosorbent assay. Receiver operating characteristic curve (ROC) was used to evaluate the diagnostic power of ANGPT2 for AAD. Transcriptome data demonstrated that a total of 18 genes were significantly up-regulated and 28 genes were significantly down-regulated among AAD tissues (foldchange>3.0, *p* < 0.01). By bioinformatic analysis, we identified ANGPT2 as a candidate biomarker for blood-based detection of AAD. The qRT-PCR and protein expression demonstrated that ANGPT2 increased 2.4- and 4.2 folds, respectively in aortic tissue of AAD patients. Immunohistochemical staining demonstrated that ANGPT2 was markedly increased in intima of the aortic wall in AAD. Furthermore, ANGPT2 was significantly elevated in AAD patients as compared with controls (median 1625 vs. 383 pg/ml, *p* < 1E-6). ROC curve analysis showed that ANGPT2 was highly predictive of a diagnosis of type A AAD (area under curve 0.93, *p* < 1E-6). Sensitivity and specificity were 81 and 90%, respectively at the cutoff value of 833 pg/ml. In conclusion, ANGPT2 could be a promising biomarker for diagnosis of AAD; however, more studies are still needed to verify its specificity in diagnosing of AAD.

## Introduction

Acute aortic dissection (AAD) represents the most common thoracic aortic emergency with a death rate of 1–2% per hour after symptom onset and approximately 50% of patients die in the first 48 h if left untreated (1). Meanwhile, the symptoms of AAD vary greatly and lack of specificity and the misdiagnosis occurs in 25–50% of patients on initial evaluation (2–4). The rapid progress, excessively high mortality and misdiagnosis possibility make prompt diagnosis urgent for AAD. Nowadays, AAD has become more identifiable owing to the increased use of advanced imaging modalities, such as computed tomography, transesophageal echocardiography and magnetic resonance imaging (5). However, these imaging tests are expensive and have limited bedside availability, especially in developing countries. Also, some patients are hemodynamically unstable at admission, making the diagnostic methods difficult to perform. Therefore, looking for proper tools that are convenient and inexpensive is of great importance for universal application.

During the past years, a series of potential diagnostic biomarkers have been reported to be related with AAD such as smooth muscle myosin heavy chain, creatine kinase BB-isozyme, calponin, elastin and D-dimer, etc (6). However, the sensitivity and (or) specificity limited their wide use as the gold standard for diagnosis of AAD in clinical practice. Therefore, a biomarker with high sensitivity and specificity for AAD is still urgently needed.

Transcriptomic strategy has been proven to be an effective way to identify new protein-markers involved in cardiovascular diseases (7–9). In this study, we applied the transcriptomic approach to search the novel circulating biomarkers for AAD. We compared gene expression profiles for aortic tissue from Standford type A AAD patients and healthy controls, and determined genes differentially expressed in the pathological situation. We further tested a promising candidate biomarker for blood-based discrimination of AAD.

## Materials and Methods

### Study Cohorts

The first patient cohort was consisted of 8 patients with Standford type A AAD who received Bentall operation from April 2012 to June 2012 at Fuwai Hospital, National Center for Cardiovascular Diseases. The median time since symptom onset to admission was 18 hours (14–32 h) and the median time from admission to operation was 26 h (17–45 h). The dissecting tissue samples of ascending aorta were obtained from patients at the time of operation. Control aortic tissues were obtained from 8 healthy organ donors without cardiovascular diseases. After being macro dissected, the aortic tissues were stored in liquid nitrogen for RNA and protein analysis, or fixed in 4% paraformaldehyde for pathological analysis.

The second cohort included 77 consecutive patients with Standford type A AAD referred to Fuwai Hospital between May 2012 to December 2012 and 82 healthy controls. The median duration from symptom onset to admission in these patients was 24 h (12–96 h). Baseline blood sample was taken from each participant and was immediately cooled on ice and centrifuged at 4°C. Plasma samples were divided into aliquots and stored frozen at −80°C until detection.

The diagnosis of AAD was based on standard criteria with confirmation by the multi-detector computed tomographic scanning. Patients were ruled out of the possibility of Marfan syndrome, Loeys-Dietz syndrome, and familial aortic dissection. This study was performed in accordance with the principle of the Declaration of Helsinki and the research protocol was approved by the institutional ethical review board of Fuwai Hospital. All participants gave written informed consents for storage of serum samples and future biomarker analyses at the time when the samples were obtained.

### Gene Expression Profiling

Whole-genome expression profiling was assayed in a panel of aortic tissues from 4 male AAD patients and 4 male healthy controls using Affymetrix HG-U133 Plus 2.0 GeneChip (Affymetrix, Santa Clara, CA). This mRNA microarray analysis was performed at Shanghai Biotechnology Corporation. Total RNA was extracted using mirVana miRNA Isolation Kit (Ambion, Austin, TX, US) and RNase-Free DNase Set (QIAGEN, GmBH, Germany) following the manufacturer's instructions. The quality and concentration of RNA was determined using the Agilent Bioanalyzer 2100 (Agilent Technologies, Santa Clara, CA, US). Only RNA that passed Bioanalyzer analysis with the RNA integrity number ≥ 7.0 and 28S/18S ≥ 0.7 was used for further analyses. Five hundred nanograms of total RNA were amplified, labeled and purified by using GeneChip 3'IVT Express Kit (Affymetrix, Santa Clara, CA, US) to obtain biotin labeled cRNA. Array hybridization and wash were performed using GeneChip Hybridization, Wash and Stain Kit (Affymetrix) in Hybridization Oven 645 (Affymetrix) and Fluidics Station (Affymetrix) by following the manufacturer's instructions. Slides were scanned by GeneChip Scanner 3000 (Affymetrix) and Command Console Software 3.1 (Affymetrix) with default settings. Raw data were normalized by MAS 5.0 algorithm, Gene Spring Software 11.0 (Agilent technologies, Santa Clara, CA, US).

### Quantitative Real-Time PCR

Expression validation was performed in the aortic RNA from 8 AAD patients and 8 controls by quantitative real-time PCR (qRT-PCR). First-strand cDNA was synthesized from 1000 ng total RNA using a mixture of both oligo-dT and random hexamers and the Superscript III transcript kit (Invitrogen, Paisley, UK) as previously described (10). Quantitative real-time PCR was carried out by using Mx3000P (Stratagene Japan Inc., Tokyo, Japan) with Brilliant II SYBR Green QPCR Master Mix (Stratagene). The primer sequences and PCR conditions were listed in Table 1. Glyceraldehyde 3-phosphate dehydrogenase (GAPDH) was analyzed in parallel as an internal control. All experiments were repeated at least twice in triplicate.

**Table 1:** Primers for quantitative real-time PCR.

**Gene symbol**	**Primer name**	**Primer sequence**	**Tm (°C)**	**Product length (bp)**
ANGPT2	ANGPT2-F	CAA GTG CTG GAG AAC ATC	55	228
	ANGPT2-R	AAG TCT CGT GGT CTG ATT TA		
CCL3	CCL3-F	CGA GCC CAC ATT CCG TCA C	55	240
	CCL3-R	CGG CTT CGC TTG GTT AGG AA		
GAPDH	GAPDH-F	CTC TGA CTT CAA CAG CGA CAC C	53	111
	GAPDH-R	TAG CCA AAT TCG TTG TCA TAC C		

### Western Blotting Analysis

The aortic tissue was lysed in RIPA buffer (50 mM Tris, pH 7.4, 150 mM NaCl, 1% Triton X-100, 1% sodium deoxycholate, 0.1% SDS, 1 mM EDTA, and protease inhibitor cocktail) using homogenization at 20,000 rpm for 1 min and centrifugation at 12,000 rpm at 4°C for 30 min. Supernatants were incubated with loading buffer in boiling water for 5 min, separated on a SDS-PAGE gel, transferred onto a nitrocellulose membrane and blocked for 1 h with 5% BSA. The membrane was incubated overnight with antibodies specific to ANGPT2 (AB623, R & D System, Minneapolis, MN), followed by incubation for 1 h with a 1:20,000 dilution of horseradish peroxidase-conjugated anti-goat IgG. The signal was visualized by enhanced chemiluminescence (Santa Cruz) and analyzed using Gel-Pro Analyzer software. Beta-actin was used for internal protein normalization.

### Immunohistochemical Staining

Formalin-fixed aortic wall tissues of healthy controls and AAD patients were routinely dehydrated and paraffin embedded. The samples were sectioned at 5 μm and stained with hematoxylin-eosin (HE), elastica van Gieson's (EVG), and Masson's trichrome for observation. To expose target proteins, the antigen was retried using 10 mM sodium citrate (pH 6.0) high pressure hot repaired for 3 min. The endogenous peroxidase was blocked with 3% H_2_O_2_ for 10 min at room temperature, then washed with ddH_2_O and PBS. ANGPT2 staining was performed using a polyclonal monospecific antibody (1:100 in 5% BSA-PBS, Rabbit anti-human ab153934, Abcam, America). Primary antibody was incubated for overnight at 4°C and reheated for half an hour at 37°C. A HRP-conjugated anti-rabbit was used as the secondary antibody (PV-6001, ZSGB-BIO, China), followed by colorimetric detection using a DAB kit (ZLI-9017, ZSGB-BIO, China). Tissues were counterstained with hematoxylin and dehydrated with ethanol and xylene to prep for mounting. Positive results of immunohistochemistry indicated yellowish brown or brown particles.

### Enzyme-Linked Immunosorbent Assay (ELISA)

The plasma concentration of ANGPT2 was determined using Human DuoSet ELISA kit (DY623 for Angiopoietin-2, R & D System, Minneapolis, MN), according to the manufacturers' instructions. The ELISA test was performed in triplicate and repeated twice on each plasma sample. The intra-assay and inter-assay coefficients of variation were less than 5 and 8%, respectively.

### Statistical Analysis

Normally distributed data were reported as mean ± SD, and non-normally distributed data were reported as median and interquartile range (25 to 75%) tested by the normal distribution analysis.

Categorical data were presented as numbers and proportion. Statistical analysis of continuous variables with equal variance and normal distribution was carried out using independent-sample *t* test. Data with un-equal variance and (or) non-normal distribution were compared with Mann-Whitney test. Chi-square test was used for categorical variables. Sensitivity and specificity were calculated in relation to the final diagnosis. Receiver operating characteristics (ROC) curves were constructed by plotting sensitivity (true-positive fraction) vs. 1-specificity (false-positive fraction) for discrimination between controls and patients with AAD. The area under the curve was calculated. A multivariable Logistic regression analysis was used to determine the association of ANGPT2 with AAD and the model adjusted for age, gender, hypertension, coronary heart disease, diabetes, hyperlipidemia, smoking and alcohol history. Statistical analyses were performed with PASW Statistics 18.0 software. In all cases, *p* < 0.05 was considered to be significant.

## Results

**Figure 1:**
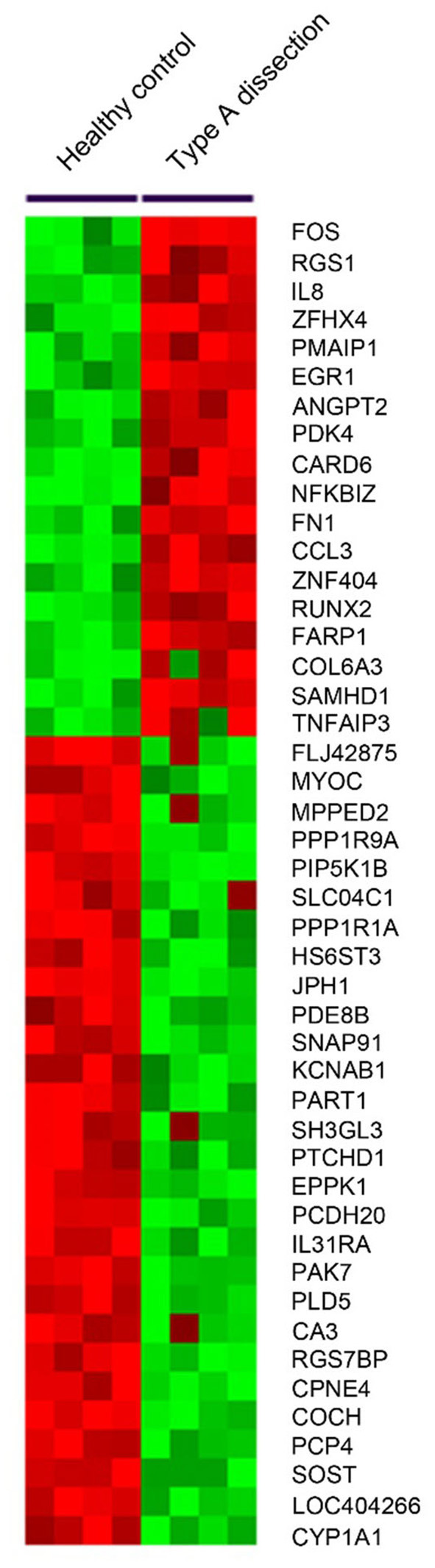
A heatmap was generated by clustering based on probes differentially expressed in dissected aortas as compared with normal aortas. The gene associated with each probe is indicated to the right of the heatmap.

To screen genes that were dysregulated in AAD, transcriptome profiles were measured in the ascending aortic tissues of 4 male patients with Standford type A AAD (mean age, 49.1 ± 4.9 years) and 4 male healthy controls (mean age, 47.9 ± 6.7 years). As the Affymetrix GeneChip contains about 38,500 human genes as represented by analysis of over 47,000 transcripts and variants, we used stringent criteria (foldchange ≥ 3 and *p* < 0.01) to reduce the chance of false-positive. A total of 46 genes were identified significantly differentially expressed in dissected aortas as compared with healthy controls. The expression differences were sufficient for proper clustering and supervised classification of control and AAD aortic samples (Figure 1). The clustering analysis also showed that groups of probes had similar patterns of AAD across the samples.

Among the 46 differentially expressed genes, 18 were up-regulated and 28 genes were down-regulated (Figure 1). Our present study aimed to explore the novel biomarkers for diagnosing AAD, therefore, secretory proteins that could be detected in peripheral blood were considered. Then the secretory annotation of the upregulated genes was analyzed using the data from Uniprot Database and Secreted Protein Database. Three genes, including interleukin-8 (IL-8), ANGPT2 and chemokine C-C motif ligand 3 (CCL3) were annotated as secretable and the cell types responsible for the secretion of these proteins were analyzed with online tool (https://data.humancellatlas.org) (Table 2). After reviewing the literature, we found that circulating IL-8 has been reported to be increased in AAD (11) while ANGPT2 and CCL3 have never been reported in AAD. Therefore, we selected ANGPT2 and CCL3 for further evaluation.

**Table 2:** List of upregulated genes in aortic tissues of Standford type A dissection.

**Gene symbol**	**Gene title**	***p* value**	**Fold change**	**Secreted**	**Possible cells for secretion**
FOS	FBJ murine osteosarcoma viral oncogene homolog	4.6E-03	7.79		
RGS1	regulator of G-protein signaling 1	9.6E-03	7.61		
IL8	interleukin 8	3.8E-03	6.69	Yes	smooth muscle cell, endothelial cell
ZFHX4	zinc finger homeobox 4	2.3E-03	4.6		
PMAIP1	phorbol-12-myristate-13-acetate-induced protein 1	2.3E-03	4.07		
EGR1	early growth response 1	3.2E-03	4.06		
ANGPT2	angiopoietin 2	6.4E-03	4.02	Yes	endothelial cell
PDK4	pyruvate dehydrogenase kinase, isozyme 4	1.8E-03	3.94		
CARD6	caspase recruitment domain family, member 6	5.2E-03	3.86		
NFKBIZ	nuclear factor of kappa light polypeptide gene enhancer in B-cells inhibitor, zeta	3.8E-03	3.83		
FN1	fibronectin 1	1.0E-03	3.68		
CCL3	chemokine (C-C motif) ligand 3	1.8E-03	3.65	Yes	smooth muscle cell, endothelial cell
ZNF404	zinc finger protein 404	3.3E-03	3.6		
RUNX2	runt-related transcription factor 2	3.0E-03	3.58		
FARP1	FERM, RhoGEF (ARHGEF) and pleckstrin domain protein 1 (chondrocyte-derived)	2.4E-04	3.52		
COL6A3	collagen, type VI, alpha 3	9.9E-03	3.38		
SAMHD1	SAM domain and HD domain 1	1.2E-03	3.19		
TNFAIP3	tumor necrosis factor, alpha-induced protein 3	9.5E-03	3		

**Figure 2:**
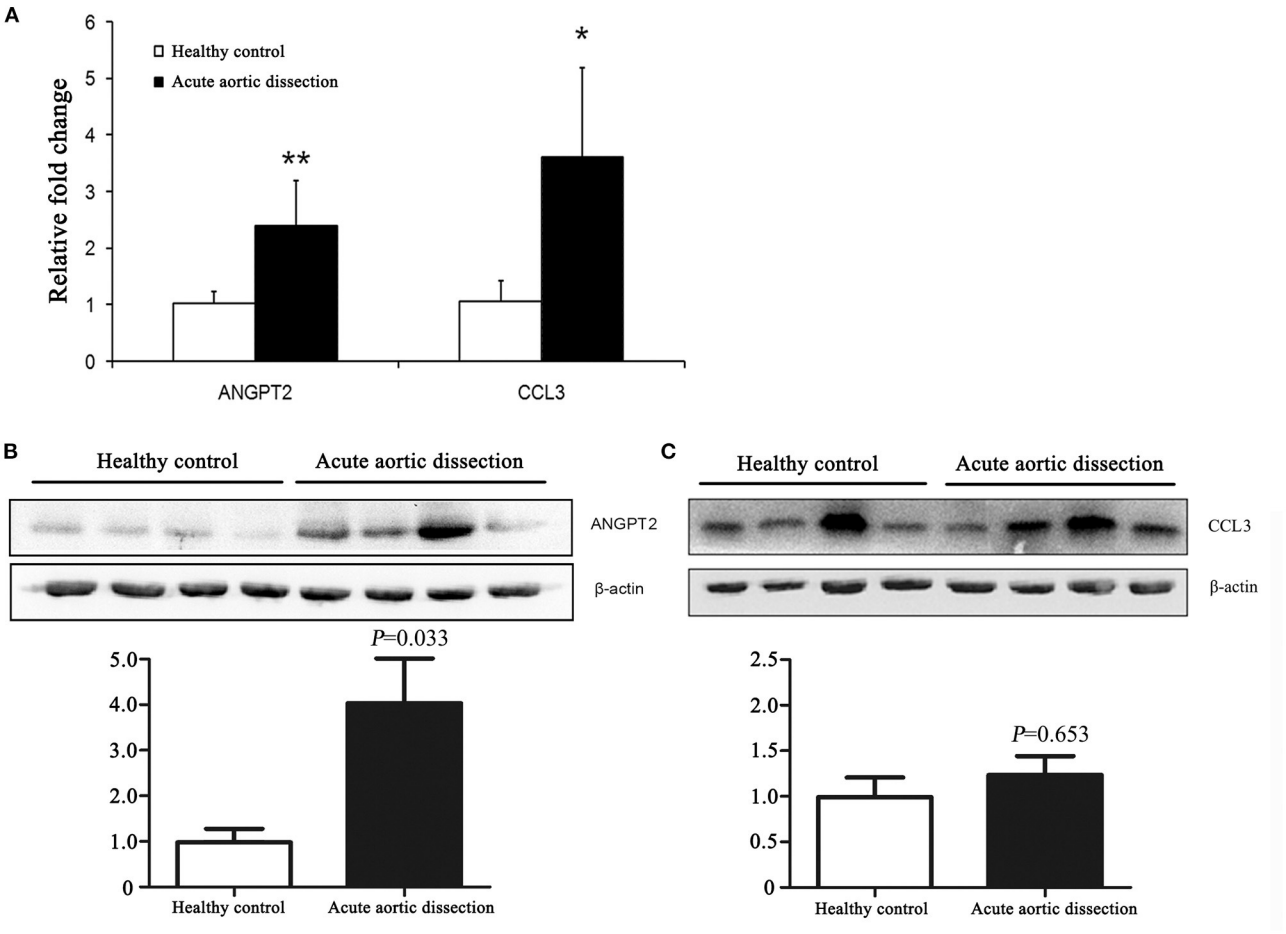
Validation of expression changes of ANGPT2 and CCL3 in transcription and protein level. **(A)**: Validation of microarray data by quantitative Real-Time PCR (qRT-PCR). The expression patterns of ANGPT2 and CCL3 increased 2.4-fold (*P* < 0.01) and 3.6-fold (*P* = 0.02) in aortic tissues of AAD compared with healthy control. GAPDH was used as housekeeping gene. ^**^: *P* < 0.01, ^*^: *P* < 0.05. **(B,C)**: Western blot for ANGPT2 and CCL3 protein from aortic tissues of healthy control and AAD patients. β-actin serves as an internal control. ANGPT2 increased up to 4.2 folds in AAD patients, whereas CCL3 did not change.

To validate the microarray results, qRT-PCR was used to determine the transcript levels of ANGPT2 and CCL3 in aortic tissues of 8 AAD patients and 8 healthy controls. As shown in Figure 2A, ANGPT2 and CCL3 increased 2.4-fold (*p* < 0.01) and 3.6-fold (*p* = 0.02), respectively in the patient group, compared with control specimens. Western blot demonstrated that protein expression of ANGPT2 also increased up to 4.2 folds in aortic tissue of AAD patients (*p* = 0.03) (Figure 2B). In contrast, the CCL3 did not change at the protein level (Figure 2C). Therefore, we focused on the ANGPT2 in the following study.

**Figure 3:**
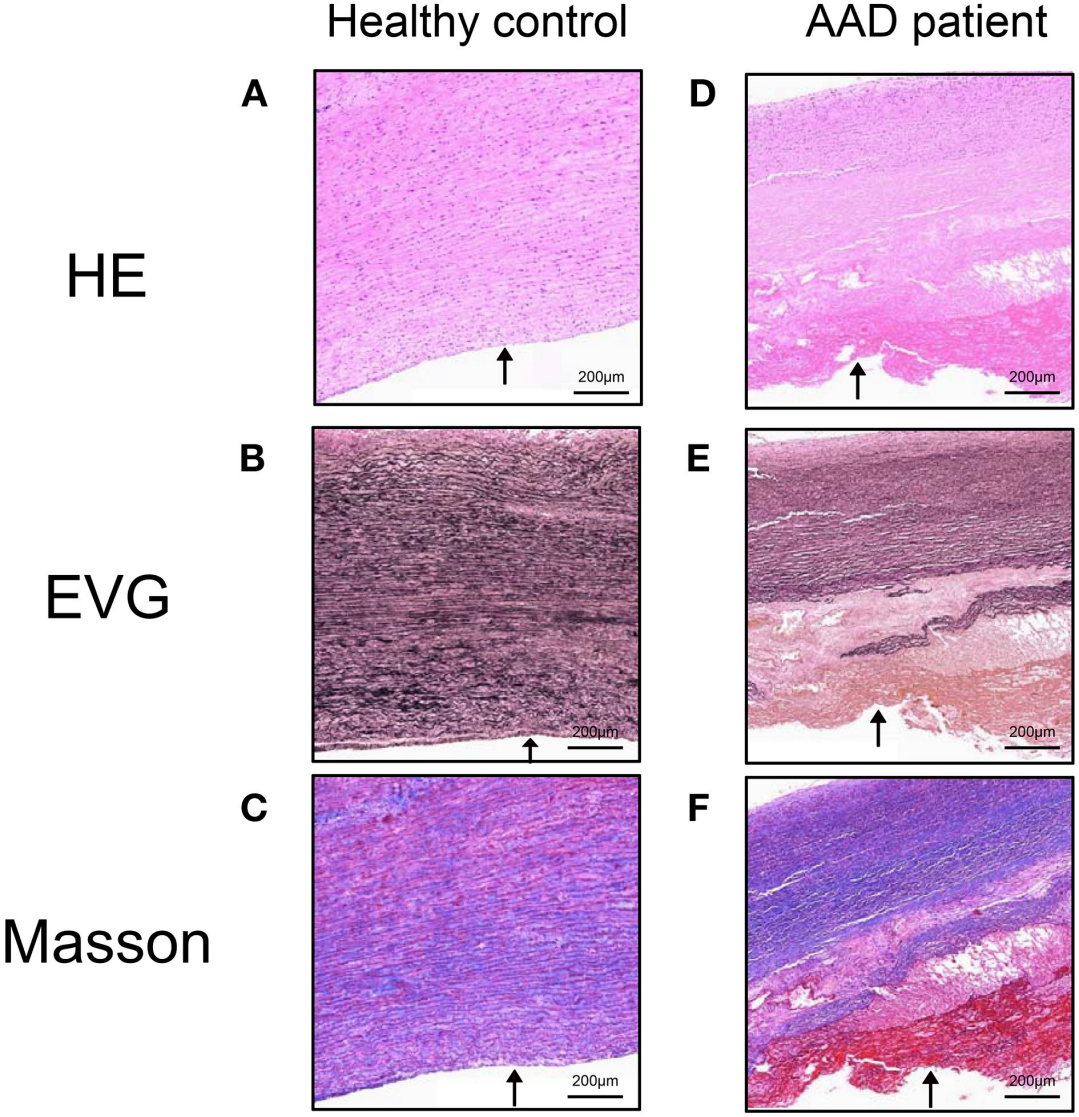
Aortic pathology of AAD patients and healthy controls. The elastic fibers were properly oriented in control aortas and laminar medial necrosis, inflammation, and giant cells were not present in healthy controls **(A–C)**. In contrast, the aorta wall tissues were loosely structured and disordered in AAD patients. The dissected aortas were found medial degeneration, as shown by loss of smooth muscle cells and elastic fibers, and proteoglycan accumulation in the medial layer of the aorta **(D–F)**. Arrows indicated the lumen side. HE, hematoxylin-eosin; EVG, elastica van Gieson's; Masson, Masson's trichrome. Magnification: ×100. AAD, acute aortic dissection.

The ascending thoracic aortic pathology was reviewed for healthy controls and AAD patients (Figure 3). The elastic fibers were properly oriented in control aortas (Figure 3B) and laminar medial necrosis, inflammation, and giant cells were not present in healthy controls (Figures 3A–C). In contrast, the aorta wall tissues were loosely structured and disordered in AAD patients. The dissected aortas were found medial degeneration, as shown by loss of smooth muscle cells and elastic fibers, and proteoglycan accumulation in the medial layer of the aorta (Figures 3D–F).

**Figure 4:**
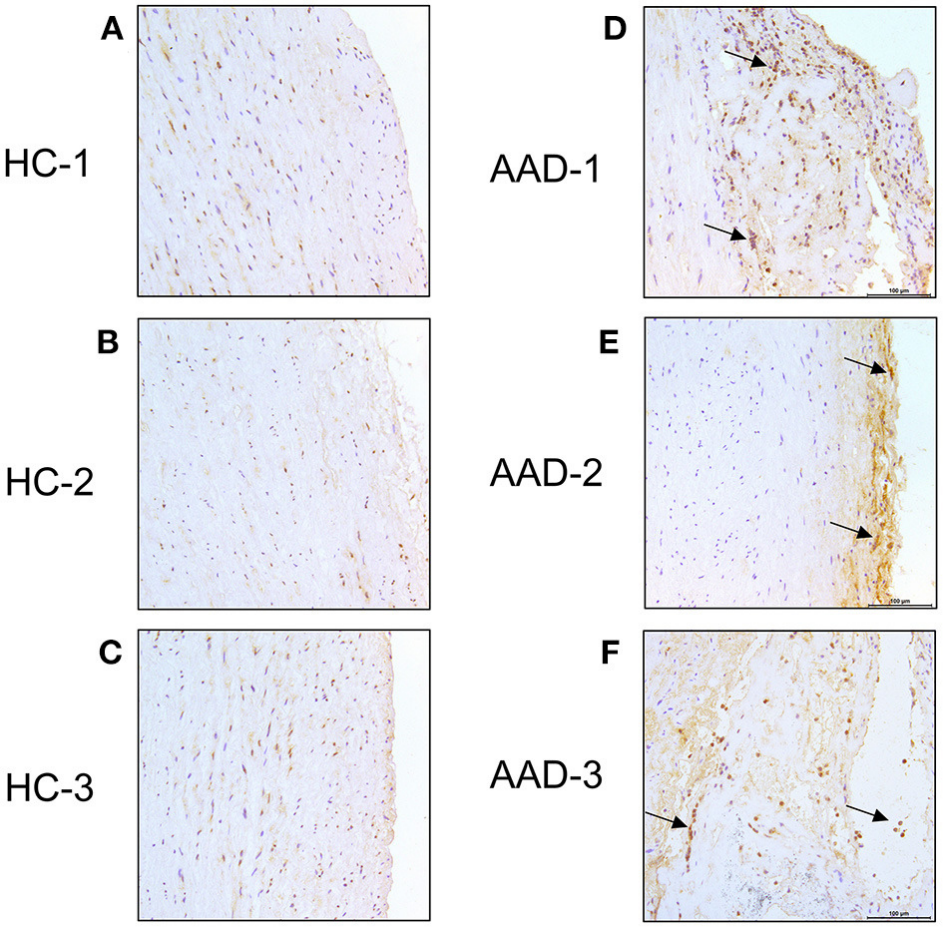
Immunohistochemical staining. Ascending aorta specimen in the entry site of the dissection from AAD patients were sectioned and labeled with ANGPT2. **(A–C)**, control aortas exhibited a weaker signal of ANGPT2. **(D–F)**, Intense ANGPT2 signals were presented in the thoracic aortic dissection patient. Arrows indicate examples of ANGPT2 positive cells. HC, healthy control; AAD, acute aortic dissection; magnification: × 200.

We then analyzed the expression of ANGPT2 in the aortic sections of healthy controls and AAD patients (Figure 4). In healthy control aortas, ANGPT2 was expressed at a very low level in the intima of the vessel (Figures 4A–C). In contrast, in aortas from AAD patients, ANGPT2 protein expression was markedly increased in intima and dissecting layers of the aortic wall (Figures 4D,E). The majority of ANGPT2 positive cells were located in deeper parts of the entry site of the dissection. In consistent with the qRT-PCR and western blotting, these immunohistochemical staining results clearly indicated that ANGPT2 was upregulated in patients with AAD.

**Figure 5:**
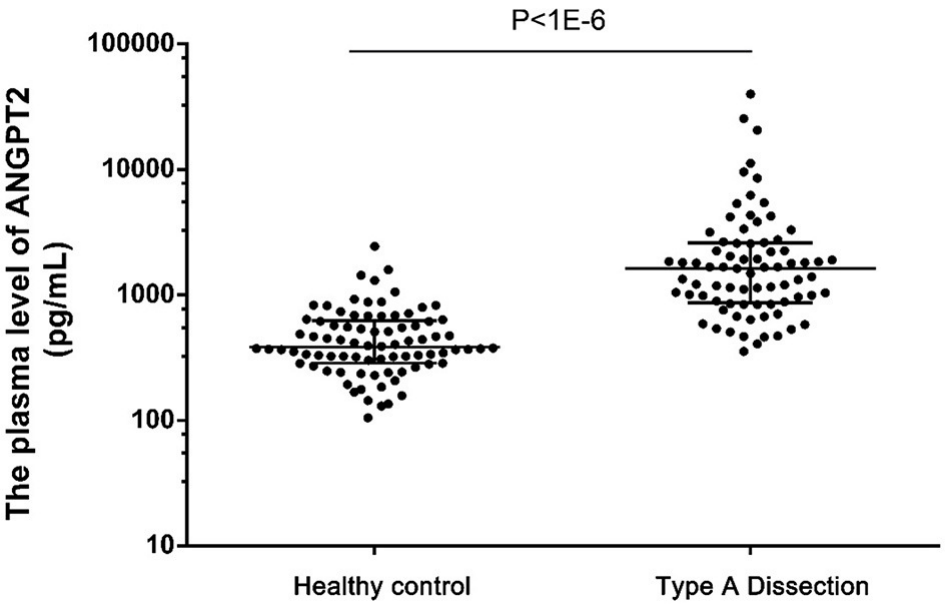
Plasma levels of ANGPT2 in healthy control (*n* = 82), Type A Dissection (*n* = 77). Data are expressed as scatter plots respresenting the median, 25 and 75 percentiles. Statistical analysis: Mann-Whitney non-parametric test.

**Figure 6:**
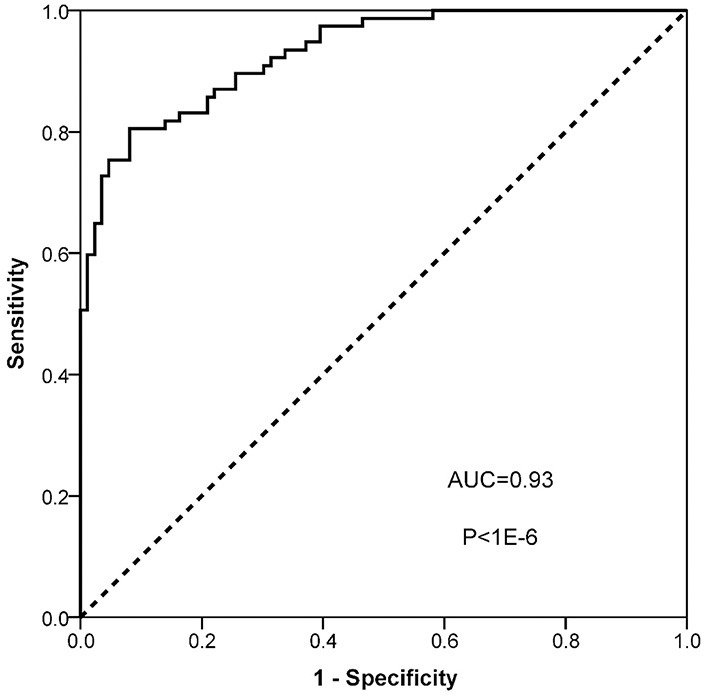
ROC analysis of ANGPT2.

To evaluate the diagnostic value of ANGPT2 in AAD, the plasma level of ANGPT2 was measured in the second cohort, which was composed of 77 patients with Standford type A AAD and 82 healthy controls. Characteristics of the cohort are shown in Table 3. No significant difference was observed regarding age and gender between the 2 groups; however, AAD patients were more likely to be smoker and had a relatively lower total cholesterol and low density lipoprotein. Individual values of ANGPT2 in AAD patients and in controls are presented in Figure 5. ANGPT2 was significantly elevated in Standford type A AAD patients (median 1625 pg/ml, interquartile range 866–2595 pg/ml) as compared with healthy controls (medians 383 pg/ml, interquartile range 285–622 pg/ml, *p* < 1E-6). ROC curve analysis showed that ANGPT2 was highly predictive of a diagnosis of type A AAD (area under curve 0.93, *p* < 1E-6). Sensitivity and specificity were 81 and 90%, respectively at the cutoff value of 833 pg/ml (Figure 6).

**Table 3:** Demographic and baseline characteristics of the validation cohort.

	**Healthy control (*n* = 82)**	**Type A dissection (*n* = 77)**	***p* value**
Age (years)	52.4 ± 9.9	52.4 ± 10.0	1.000
Male (%)	45 (54.9%)	53 (68.8%)	0.075
Hypertension (%)	39 (47.6%)	48 (62.3%)	0.079
Diabetes (%)	2 (2.4%)	0 (0.0%)	0.497
Coronary heart disease (%)	2 (2.4%)	5 (6.5%)	0.265
Smoking (%)	20 (24.4%)	46 (59.7%)	<0.001
Drinking (%)	18 (22.0%)	16 (20.8%)	1.000
Systolic blood pressure (mmHg)	130 (120–150)	134 (120–155)	0.462
Diastolic blood pressure (mmHg)	80 (65–90)	80 (70–90)	0.502
Heart rate (beats/min)	82 (76–90)	80 (71–90)	0.608
Creatinine (umol/L)	87.5 (78.5–101.4)	94.8 (78.4–117.2)	0.095
Total cholesterol (mmol/L)	3.9 (3.1–4.7)	3.4 (3.0–3.9)	0.002
Triglyceride (mmol/L)	1.3 (1.0–1.6)	1.2 (1.0–1.6)	0.302
Low density lipoprotein (mmol/L)	1.9 (1.4–2.7)	1.8 (1.4–2.1)	0.042
High density lipoprotein (mmol/L)	0.9 (0.7–1.0)	0.9 (0.7–1.1)	0.522

The correlation of ANGPT2 with other clinical parameters associated with AAD was further analyzed (Table 4). It was found ANGPT2 was only correlated with the creatinine (spearman correlation coefficient 0.736, *p* < 0.001). Then a multivariable regression analysis was used to evaluate the independent factors for type A AAD. After adjusting for age, gender, hypertension, coronary heart disease, diabetes, hyperlipidemia, smoking and alcohol history, It was found ANGPT2 level > 833 pg/ml was independently associated with type A AAD (RR = 47.297, 95%CI 16.497-135.601, *p* < 0.001).

**Table 4:** Spearman correlation coefficients between ANGPT2 and other clinical variables.

**Variables**	**ANGPT2**	***p* value**
Age	−0.134	0.244
Leukocyte counts	−0.026	0.821
D-dimer	−0.108	0.361
Ascend aortic diameter	0.021	0.861
Creatinine	0.736	<0.001
C reactive protein	−0.061	0.681

## Discussion

Although some biomarkers are commonly used for assisting for AAD diagnosis, the sensitivity and (or) specificity of these biomarkers are not satisfactory, therefore limited their diagnostic power in clinical practice. For instance, D-dimer is regarded as the most useful marker to provide information for diagnosing AAD, although the sensitivity is as high as 0.952, the specificity is only 0.604 (12). Therefore, additional more accurate biomarkers are needed for improved diagnosis based on a clearer understanding of the underlying pathophysiology of AAD.

Affymetrix microarray is the most widely used high-throughput technology to measure gene expression analysis (13) and has successfully identified many biomarkers for molecular mechanisms, early diagnosis and prognostic evaluation in many diseases (14–17). The most distinguishing feature of Affymetrix GeneChip microarray is that their manufacture is by using light-controlled *in situ* synthesis of DNA microarrays. This manufacturing technology allows the inclusion of multiple probes to interrogate the same target sequence and a large number of features to be synthesized on one array and provides accurate and high-throughput analysis of the assay. In addition, Affymetrix GeneChip microarrays cover almost all transcripts annotated in genebank. The accuracy and reproducibility of this method were show to be satisfactory. We used Affymetrix microarrays to identify differentially expressed mRNA from AAD-diseased and normal aorta which guaranteed the sensitivity and specificity of our biomarkers. We have found the differentially expressed genes are supposed to be associated mainly with two pathways, the focal adhesion pathway and actin cytoskeleton pathway by bioinformatics analysis (18).

The ideal biomarkers for assisting diagnosing AAD are secretory and can be detected in plasma. In our present study, a differentially expressed gene, ANGPT2 was confirmed to be elevated in both aorta tissue and plasma in patients with AAD. Angiopoietins are a family of secreted glycoproteins with important roles in vascular development and angiogenesis. Angiopoietin-1 and ANGPT2 are the best known members which bind with similar affinity to tyrosine-protein kinase receptor Tie-2 (19). Angiopoietin-1 is the major agonist for Tie-2, thus inhibiting endothelial permeability and contributing to blood vessel maturation and stability; in contrast, ANGPT2 is exclusively produced by endothelial cell and acts as an antagonist for Angiopoietin-1 and Tie-2, thus disrupting the vascular remodeling ability of Angiopoietin-1 (20).

ANGPT2 has been confirmed to be upregulated in a wide range of cardiovascular diseases such as acute coronary syndrome (21), heart failure (22) and hypertension (23) etc. Moreover, ANGPT2 has also been shown to relate with the outcome in patients with cardiovascular diseases (24–26). The roles of ANGPT2 in cardiovascular disease may be context-dependent. Angiogenesis is involved in cardiovascular diseases (27) and this process requires a complex cross-talk between numerous cell types and growth factors, among which ANGPT2 provides a possible link between blood vessel formation and inflammation (28). A certain physiological ANGPT2 level or transient upregulation of ANGPT2 can stimulate revascularization while supraphysiological level or excessive duration of ANGPT2 exposure could promote tissue inflammation and vessel disorganization, thus promoting vessel regression and vascular leakage (29).

To the best of our knowledge, the present study is the first to investigate the plasma ANGPT2 level in AAD patients. Compared with the normal control, plasma ANGPT2 level in AAD patients were significantly elevated which indicated that inflammation, oxidative stress, and endothelial dysfunction might be involved in the pathogenesis of AAD. Indeed, previous studies have shown inflammatory mechanisms contribute greatly to aortic wall remodeling presented with activated T cells and macrophages in aortic specimens in patients with aneurysms or dissections (30) and a series of inflammatory makers such as C-reactive protein, interleukin-6, IL-8, interleukin-10, tumor necrosis factor-α and natural killer, B cells were elevated in the peripheral blood of AAD patients (31). Meanwhile, morphological changes in endothelial cell were also observed in patient with AAD such as hyperplasia of endothelial cell with loose cellular junctions and desquamation of endothelial cell in aortic segment (32), indicating the endothelial cell impairment in AAD, which is a possible cause of ANGPT2 release. In addition, impaired vasa vasorum flow can cause increased aortic stiffness and produce interlaminar shear stresses, leading to the development of AAD (33). The decreased vasa vasorum flow can also lead aortic wall to be in the state of hypoxia and exposure of endothelial cells to hypoxia results in increase of secreted ANGPT2 levels (34). What's more, blood flow through the non-endothelialized false lumen is a powerful activator of the hemostatic system (35) and stimulation of endothelial cell with thrombin can induce the rapid release of ANGPT2 which is stored in Weibel-Palade bodies (36). Moreover, intimal tear can confer direct injury to endothelial cell which can also induce the release of ANGPT2 in endothelial cell. The above mechanisms may partially explain the elevation of plasma ANGPT2 in AAD patients.

Our present study has several strengths in clinical practice. Firstly, there is lack of optimal biomarker for AAD although several biomarkers have been applied in daily practice. Our present study demonstrated ANGPT2 may potentially serve as a biomarker for diagnosing AAD because ANGPT2 was shown to be independently associated with AAD in our study, indicating its potential diagnostic value for AAD. However, we found ANGPT2 correlated with the creatinine level, consistent with previous findings (37), suggesting that when using this marker, other confounding factors should be considered. Secondly, up to now the precise mechanisms of AAD remain to be clarified and our present study indicated ANGPT2 may involve in the pathopoiesis of AAD. However, given its complex function in different physiological or pathological conditions, more studies are needed to elucidate the role of ANGPT2 in AAD. If ANGPT2 is confirmed to involve in pathopoiesis of AAD, whether it can become a potential therapeutic target also deserve further study. In addition, some studies have shown ANGPT2 is prognostic biomarker in cancers (38, 39), and whether ANGPT2 is associated with the outcome or can be used to risk stratification in patients with AAD is also needed to be confirmed.

Although the present study indicated plasma ANGPT2 was a promising biomarker for AAD diagnosis, some limitations also existed. The first and also the most important limitation is that we cannot demonstrate the specificity of ANGPT2 to diagnose AAD from other diseases, especially in patients with chest pain mimicking AAD such as acute myocardial infarction or pulmonary embolism. However, currently, we are performing a study with the aim to test the specificity of AGNPT2 for diagnosing AAD among different diseases and we hope we will answer this question in the future. Secondly, although we demonstrated ANGPT2 was significantly elevated in patients with AAD, whether it is elevated in chronic aortic dissection or be can used to differentiate acute or chronic dissection remains unclear. Therefore, whether it has potential differential diagnostic value for acute vs. chronic aortic dissection deserves further study. Thirdly, in our present study, we only focused on the secretory proteins differentially expressed between AAD patients and the control, some unsecretory proteins can also be released along with cell injury and tissue disruption in AAD, and they are also the potential biomarkers for AAD. Therefore, the unsecretory proteins are also promising. Fourth, the specific cell type that produces ANGPT2 was not clearly displayed in our immunohistochemical staining although it is usually expressed by endothelial cell, and whether other cell types can secret ANGPT2 in the setting of AAD also needs further study. In addition, our present study only demonstrated the association of ANGPT2 with AAD not the causal relationship. Therefore, whether aorta with high expression of ANGPT2 increases the risk of aortic dissection needs clinical perspective studies or animal experiments to confirm. Furthermore, patients in our present study were all Standford type A AAD, whether ANGPT2 has similar diagnostic value in Standford type B AAD patients warrants further study. The last but not the least, whether ANGPT2 can become an ideal molecular therapeutic target for AAD also need to be confirmed.

## Conclusion

ANGPT2 could be a promising biomarker for diagnosis of AAD; however, more studies are still needed to verify its specificity in diagnosing of AAD.

## Data Availability Statement

The original contributions presented in the study are publicly available. This data can be found here: https://www.ncbi.nlm.nih.gov/geo/query/acc.cgi?acc=GSE190635.

## Ethics Statement

The studies involving human participants were reviewed and approved by The Institutional Ethical Review Board of Fuwai Hospital. The patients/participants provided their written informed consent to participate in this study.

## Author Contributions

XF and XW designed this study. BH, LT, ZC, and LZ performed the project. WS and TL collected and analyzed the data. YY and RH conducted project administration and supervision. BH and XW processed writing-original draft. All authors commented on and approved the final manuscript.

## Funding

This work was supported by the National Natural Science Foundation of China [82170408, 81770479, and 81870050]; the Drug Innovation Major Project [2018ZX09711001-003-012]; CAMS Fund for Key Laboratory of Pulmonary Vascular Medicine [2017PT32016].

## Conflict of Interest

The authors declare that the research was conducted in the absence of any commercial or financial relationships that could be construed as a potential conflict of interest.

## Publisher's Note

All claims expressed in this article are solely those of the authors and do not necessarily represent those of their affiliated organizations, or those of the publisher, the editors and the reviewers. Any product that may be evaluated in this article, or claim that may be made by its manufacturer, is not guaranteed or endorsed by the publisher.

## Abbreviations

AAD: acute aortic dissection
ANGPT2: angiopoietin 2
CCL3: C-C motif ligand 3
IL-8: interleukin-8
qRT-PCR: quantitative reverse transcriptase PCR
ROC: receiver operating characteristic curve.
